# Building Greener Cities Together: Urban Afforestation Requires Multiple Skills to Address Social, Ecological, and Climate Challenges

**DOI:** 10.3390/plants14030404

**Published:** 2025-01-29

**Authors:** Raffaello Resemini, Chiara Geroldi, Giulia Capotorti, Andrea De Toni, Francesco Parisi, Michele De Sanctis, Thomas Cabai, Micol Rossini, Luigi Vignali, Matteo Umberto Poli, Ermes Lo Piccolo, Barbara Mariotti, Andrea Arcidiacono, Paolo Biella, Erica Alghisi, Luciano Bani, Massino Bertini, Carlo Blasi, Francesca Buffi, Enrico Caprio, Stefano Castiglione, Patrizia Digiovinazzo, Olivia Dondina, Giuliano Fanelli, Francesco Ferrini, Valentina Fiorilli, Gianluca Gaiani, Daniela Gambino, Andrea Genre, Bruno Lasserre, Alberto Maltoni, Marco Marchetti, Chiara Montagnani, Marco Ottaviano, Cinzia Panigada, Silvia Ronchi, Stefano Salata, Fabio Salbitano, Enrico Simoni, Soraya Versace, Maria Chiara Pastore, Sandra Citterio, Massimo Labra, Rodolfo Gentili

**Affiliations:** 1 Department of Earth and Environmental Sciences, University of Milano-Bicocca, 20126 Milan, Italy; micol.rossini@unimib.it (M.R.); luigi.vignali@unimib.it (L.V.); luciano.bani@unimib.it (L.B.); patrizia.digiovinazzo@unimib.it (P.D.); olivia.dondina@unimib.it (O.D.); chiara.montagnani@unimib.it (C.M.); or cinzia.panigada@rurall.it (C.P.); sandra.citterio@unimib.it (S.C.); 2 Department of Architecture and Urban Studies, Politecnico di Milano, 20133 Milan, Italy; chiara.geroldi@polimi.it (C.G.); andrea.detoni@polimi.it (A.D.T.); thomas.cabai@polimi.it (T.C.); matteo.poli@polimi.it (M.U.P.); andrea.arcidiacono@polimi.it (A.A.); daniela.gambino@mail.polimi.it (D.G.); silvia.ronchi@polimi.it (S.R.); stefano.salata@polimi.it (S.S.); mariachiara.pastore@polimi.it (M.C.P.); 3 Department of Environmental Biology, University La Sapienza of Rome, 00185 Rome, Italy; giulia.capotorti@uniroma1.it (G.C.); michele.desanctis@uniroma1.it (M.D.S.); francesca.buffi@uniroma1.it (F.B.); giuliano.fanelli@uniroma1.it (G.F.); 4 Department of Biosciences and Territory, Forestry LABs, University of Molise, 86090 Pesche, Italy; francesco.parisi@unimol.it (F.P.); lasserre@unimol.it (B.L.); ottaviano@unimol.it (M.O.); soraya.versace@unimol.it (S.V.); 5 Department of Agriculture, Food, Environment and Forestry (DAGRI), University of Florence, 50144 Florence, Italy; ermes.lopiccolo@unifi.it (E.L.P.); barbara.mariotti@unifi.it (B.M.); francesco.ferrini@unifi.it (F.F.); alberto.maltoni@unifi.it (A.M.); 6 ZooPlantLab, Department of Biotechnology and Biosciences, University of Milano-Bicocca, 20126 Milan, Italy; paolo.biella@unimib.it (P.B.); massimo.labra@unimib.it (M.L.); 7 Ente Regionale per i Servizi all’Agricoltura e alle Foreste (ERSAF), 20124 Milan, Italy; erica.alghisi@ersaf.lombardia.it (E.A.); massimo.bertini@ersaf.lombardia.it (M.B.); gianluca.gaiani@ersaf.lombardia.it (G.G.); enrico.simoni@ersaf.lombardia.it (E.S.); 8 Interuniversity Research Center “Biodiversity, Ecosystem Services and Sustainability” (CIRBISES), University La Sapienza of Rome, 00185 Rome, Italy; carlo.blasi@uniroma1.it; 9 Department of Life Sciences and Systems Biology, University of Turin, 10123 Turin, Italy; enrico.caprio@unito.it (E.C.); valentina.fiorilli@unito.it (V.F.); andrea.genre@unito.it (A.G.); 10 Department of Chemistry and Biology “A. Zambelli”, University of Salerno, 84084 Salerno, Italy; scastiglione@unisa.it; 11 Department of Architecture and Design, University of La Sapienza of Rome, 00196 Rome, Italy; ma.marchetti@uniroma1.it; 12 Rurall S.p.A.—Rural & Urban Digital Hub, 25123 Brescia, Italy; 13 Department of Agricultural Sciences, University of Sassari, 07100 Sassari, Italy; fsalbitano@uniss.it

**Keywords:** urban afforestation, ecosystem services, biodiversity, climate change, remote sensing, ecological connectivity, urban planning, landscape design, EU Nature Restoration Law

## Abstract

Urban afforestation is increasingly regarded as a key strategy for fostering biodiversity to restore and enhance the ecosystem services needed to counteract the effects of climate change in built-up areas. In Italy, several experimental afforestation projects have been launched as part of the National Recovery and Resilience Plan (NRRP), focusing on cities or metropolitan areas such as Milan, Rome, Pistoia and Campobasso. These projects follow a multidisciplinary approach, integrating botanists, foresters, urban planners, landscape architects and remote sensing specialists. The goal is to address the challenging complexity of urban forest restoration through reforestation and afforestation actions. Key innovations include the integration of transdisciplinary methodologies (landscape analysis, landscape design, forest and plant ecology) with the application of advanced remote sensing technologies and participatory community engagement frameworks to address ecological and social challenges. Experimental plots have been set up across various urban areas, testing a range of planting schemes to maximise climate change resilience and ensure long-term ecological sustainability. Emphasis has been placed on selecting drought-tolerant and thermophilic species that are better adapted to widespread warming and local urban heat islands. ‘Biodiversity strips’ with perennial flowers for insects, shrubs with berries for birds and nests for wild bees and vertebrates have been set up to enhance biodiversity in new afforestation areas. Advanced monitoring tools, such as Light Detection and Ranging (LiDAR) and multi-sensor drones, have been employed alongside field observations to assess forest growth, species survival, structural complexity and biodiversity enhancement over time. Historical analyses of landscape patterns and ecological connectivity over the past 200 years, along with evaluations of afforestation projects from the last 70 years, have provided critical insights into the successes and challenges of previous interventions, serving as a guide for future efforts. By focusing on ecological connectivity, the integration of afforested areas into the urban matrix, and citizen engagement, the current project aims to align urban forestry efforts with sustainable development goals. This comprehensive project framework addresses environmental restoration and the social and aesthetic impacts on local communities, contributing to the overall resilience and well-being of urban and peri-urban ecosystems.

## 1. Introduction

Nature-based Solutions (NBSs) are defined as ‘actions to protect, conserve, restore, sustainably use and manage natural or modified terrestrial, freshwater, coastal and marine ecosystems, address social, economic and environmental challenges effectively and adaptively, while simultaneously providing human well-being, ecosystem services and resilience and biodiversity benefits’ [1]. NBSs are being used more and more to design cities and landscapes that support economic growth while also benefiting the environment and people. Green roofs and urban forests are the most extensively studied types of NBSs [2]. Among NBSs, urban forests and afforestation programmes in and around cities are addressed as critical factors generating co-benefits and substantially contributing to urban resilience [3]. Particularly, in recent years, several afforestation initiatives have been implemented in urban areas as promising strategies to mitigate/adapt to climate issues and promote biodiversity in built-up areas, enhancing the provision of ecosystem services and increasing human well-being and social cohesion [4]. In this direction, efforts towards urban afforestation worldwide have encompassed a variety of approaches, including raising awareness through educational campaigns, fostering research and collaboration between research institutions and stakeholders, ensuring active public engagement and providing equitable access to green spaces [5]. However, most available initiatives have ultimately been directed towards the number of trees planted, often neglecting or underestimating other crucial ecological and socioeconomic principles and targets.

Afforestation programmes analysed in the present study are integral to urban forestry, a concept originating in the 19th century and revitalised in the 1960s due to its role in enhancing urban sustainability and liveability [6]. Urban forestry encompasses all trees and wooded areas within urban settings and incorporates the framework of urban and peri-urban forestry, which emphasises diverse structures and management strategies [6]. Historically influenced by cultural and regional factors, urban forestry reflects evolving environmental, social, and economic priorities.

Urban silviculture, a subset of urban forestry, bridges afforestation initiatives and sustainable urban forest management. It adapts traditional silvicultural practices to urban contexts, addressing forested patches within cities. These areas, often overlooked in urban management frameworks, require tailored strategies due to the unique socio-environmental pressures of urbanisation [7,8]. Urban silviculture emphasises systemic, interdisciplinary approaches, integrating design and other fields to adapt management to urban socioecological conditions [7,9,10,11]. Unlike conventional forestry, urban silviculture focuses on altered stand dynamics and specific challenges posed by urbanisation, necessitating modifications or new guidelines. It provides a systematic framework to apply sustainable forest management, highlighting the co-benefits of afforestation in urban environments.

Key factors such as species’ ecological traits and consistency with local natural forests have frequently been disregarded [12]. On the contrary, the restoration approach applied to afforestation programmes specifically aims at ensuring the effective provision of ecosystem services. Therefore, it requires the assessment of soil type and quality, the selection of appropriate plant species and the implementation of landscape planning based on ecological criteria to connect reforestations to the mature/natural forests surrounding urban areas [13,14,15,16]. The creation of many reforested areas remains disconnected from urban surroundings and fails to engage citizens effectively, limiting people’s perception of the many values of restored ecosystems, including recreational ones [17,18]. Urban areas pose unique challenges such as limited space, potential soil contamination, compacted surfaces, altered hydrological processes and competing land uses. Addressing these challenges requires creative solutions, participatory processes and specific management strategies [18,19,20]. Additionally, urban forestry policy and planning procedures have often overlooked scientific evidence based on ecological principles [17,21].

Therefore, it is crucial to facilitate science-based development and technical assistance for afforestation projects, integrating urban forestry into development plans and promoting partnerships and knowledge sharing among professionals and institutions. Further, it is essential to recognise that afforestation projects across cities require tailored strategies [5].

This perspective work presents a conceptual–methodological framework detailing the implementation and state of the art of a scientific afforestation plan based on an ongoing national multilevel, cooperative, and interdisciplinary project in Italy.

**Figure 1:**
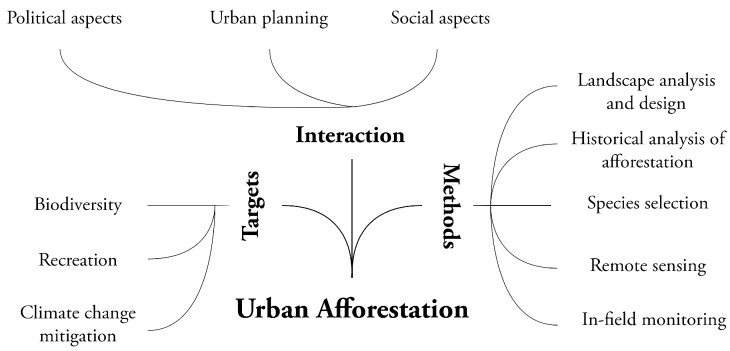
Multidisciplinary approach needed in afforestation programmes across urban environments. The diagram highlights key targets (biodiversity, recreation, climate change mitigation) and methods (landscape design, historical analysis, species selection, remote sensing and monitoring) and their interactions with urban planning, social and political aspects. This integrated approach supports sustainable and resilient urban ecosystems. (Diagram by T. Cabai).

As part of a National Recovery and Resilience Plan (NRRP) research project, a multidisciplinary team comprising architects, urban planners, landscape architects, botanists, foresters, geomorphologists, pedologists and remote sensing experts has been established to address afforestation challenges across urban areas. The project primarily aims to inform future afforestation programmes in Italy by conducting experimental plantations, integrating landscape architecture and urban design and planning, alongside advanced monitoring of past and current plantations using remote sensing techniques (mobile laser scanners, multi-sensor drones and satellites) (Figure 1).

At the international level, many guidelines for afforestation, reforestation or tree planting have been produced especially to address ecological issues, promoting biodiversity-friendly practices and responding to global challenges of climate change mitigation [22,23]. However, contributions describing multidisciplinary approaches involving teams with quite different competencies (e.g., architects, foresters, etc.) are lacking.

Past afforestation actions over the last century were considered in this project to assess the strengths and weaknesses of interventions, dynamic trends and the status of forest ecosystems over different periods. Given the highly dynamic behaviour of new urban forests, both in space and time, the effects of forest restoration in disturbed areas are best assessed by adopting landscape ecology techniques [24].

These activities were conducted in various Italian cities and metropolitan areas (including Campobasso, Milan, Pistoia and Rome) across different ecoregions [25] and with varied urban sizes and features. Following a comprehensive assessment of existing afforested patches within urban areas, experimental plots were established to evaluate the outcomes of various plantation schemes. Simultaneously, the team assessed the landscape framing and ecological connectivity of the targeted areas over the past 200 years, as well as their integration into the urban context, to better guide future planning and policy. Monitoring will continue for a minimum of three years, employing a set of innovative indicators and utilising drones equipped with multiple sensors (such as LiDAR).

This multilevel, interdisciplinary approach aims to guide future afforestation efforts by applying restoration principles that effectively contribute to achieving Sustainable Development Goals (SDGs) (e.g., Goals 11, 13 and 15) [26].

## 2. Methodological Framework

This research project primarily aims to test the effectiveness of forest restoration techniques in terms of biodiversity and forest establishment and structure in urban and peri-urban areas across four Italian cities (or metropolitan areas), namely Campobasso, Milan, Pistoia and Rome, with different sizes and geographic settings (Table 1).

**Figure 2:**
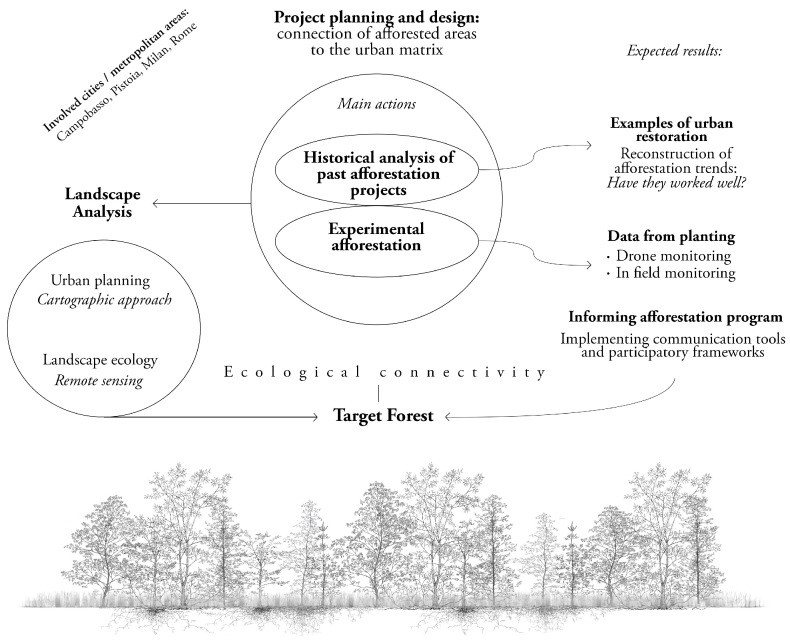
The main actions and the workflow used to achieve the main results and forest restoration across urban areas in Italian cities/metropolitan areas. (Diagram by T. Cabai).

Forest restoration will be carried out by preliminary studying and monitoring vegetation dynamics triggered by past and present afforestation actions. The main actions of the project are as follows (Figure 2):

(1) Historical analysis of past afforestation projects—This key step involves collecting and analysing data from plantings over the last 70 years. The success of the interventions will be assessed ex post, considering the age of planting, species used and proximity to the target vegetation of ancient forest patches and ‘natural’ formations as a reference baseline;
(2) Experimental afforestation—This action aims to identify the best-performing planting schemes and species to cope with climate change. In particular, several planting techniques will be tested, considering (a) the dominance of shrub species in plantations (lower water requirements); (b) the successional stages of vegetation (maximum density of trees and shrubs); (c) the use of more thermophilous species than those commonly used;
(3) Informing future afforestation programmes—This activity will be conducted to inform and involve stakeholders (e.g., administrations, citizens and the scientific community) on how to face afforestation actions, specifically using a multidisciplinary approach, integrating different forms of expertise to better protect the future forests. Effective communication tools and participatory frameworks will be used to foster collaboration, ensuring evidence-based decision-making and promoting long-term ecological and social benefits.

To achieve forest restoration, we will follow the workflow outlined in the subsequent chapters:

- Landscape analysis and ecological connectivity;
- Strategy and project concepts: connecting afforested areas to urban contexts;
- Experimental afforestation framework and species choice;
- Monitoring of afforestation interventions.

## 3. Landscape Analysis and Ecological Connectivity

### 3.1. Historical Contextualisation

**Figure 3:**
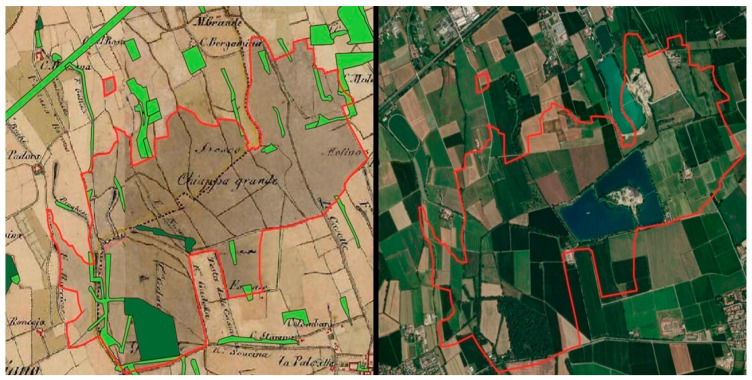
Example of forested area mapping from the historical cadastre and respective present-day remnants (Chiappa grande forest, Municipality of Cusago, Metropolitan City of Milan). On the **left**: a portion of the Habsburg cadastre (credits: Arcanum Adatbázis Kft., Budapest, Hungary) with overlaid forest remnants from 2021; the grey colour in the cadastre represents the ancient forest cover, the dark green the relict forest zone, and the light green recent woodlands or hedgerows. On the **right**: a satellite image showing remnants in 2021, within the same area identified as forested in the cadastre, delimited in red. (Data processing and pictures by A. Arcidiacono, A. De Toni, S. Ronchi, S. Salata).

To successfully guide future afforestation interventions, it is crucial to critically analyse landscape processes and history to better understand the present Land Use and Land Cover (LULC) characteristics, ultimately setting future planning and policy priorities. To this end, historical cadastres and current maps are useful for analysing landscape patterns and their change over time [29,30], as well as considering the impact that urban and landscape plans have had on LULC features and landscape composition. Specifically, cadastral maps were analysed (e.g., for the City of Milan, in the Habsburg cadastre—the second military survey of the Habsburg Empire of 1818–1829 considering the metropolitan City of Milan) (Figure 3) and the landscape structure compared to the current LULC maps (year 2021), calculating landscape metrics for different LULC classes in the two different time steps and following the methodology already applied in various studies [30,31]. Unlike the approaches and results of previous research, the results of the landscape structure evolution analysis will be related to the identification of forest remnants (i.e., unchanged forest as an ecological reference state) and recent afforested areas from different LULCs [32] to support the monitoring of the intervention phase of analysis. On the other hand, the results will also be analysed considering the implementation of historic and current urban plans to ultimately highlight how policies and plans have affected landscape structure changes and how such practices can be improved in the future to maintain and preserve biodiversity, reverse the degradation of ecosystems and generally support the provision of ecosystem services, reversing the degradation of ecosystems as required by the EU Biodiversity Strategy for 2030 [33] and EU Nature Restoration Regulation [34].

### 3.2. Current Ecological Connectivity and Ecosystem Service Provision

The analysis of current ecological connectivity and ecosystem service provision is essential to guide future afforestation efforts. Indeed, analysing and maintaining landscape connectivity is fundamental for several ecological processes [35], including dispersal and gene flow, as well as identifying anthropogenic barriers and reducing their negative impacts, considering the heightened competition between land take and ecological conservation in urban settings [36], which fragment ecological spaces, diminishing ecosystem service provision [37,38]. Thus, in each study area, the current landscape-scale ecological network was analysed, identifying its main features such as core areas, buffer areas, defragmented urban areas to be maintained and main anthropogenic threats to contextualise afforestation interventions within a broader framework.

## 4. Strategy and Project Concepts: Connecting the Afforested Areas to Urban Contexts

**Figure 4:**
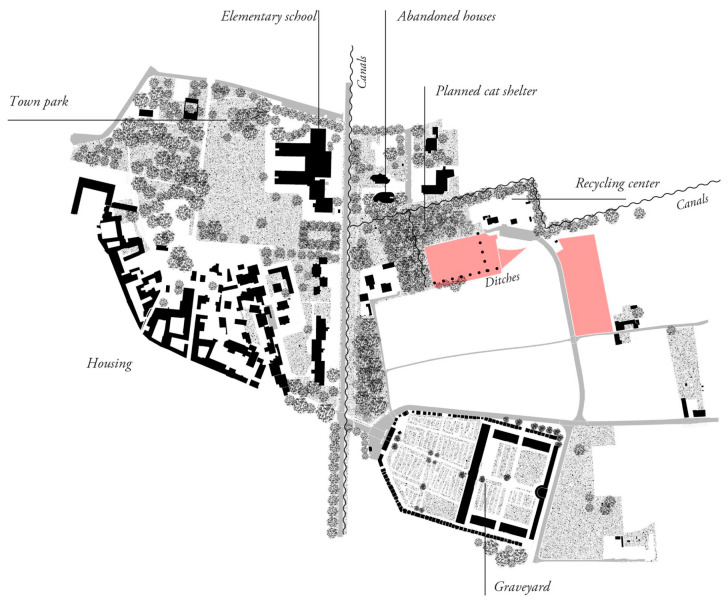
Example of a study of the relationship between the urban context and the proposed composition of the plots in the Municipality of Corbetta with the relevant project areas highlighted in pink. (Drawings by the landscape architecture research team C. Geroldi, T. Cabai, M.U. Poli).

**Figure 5:**
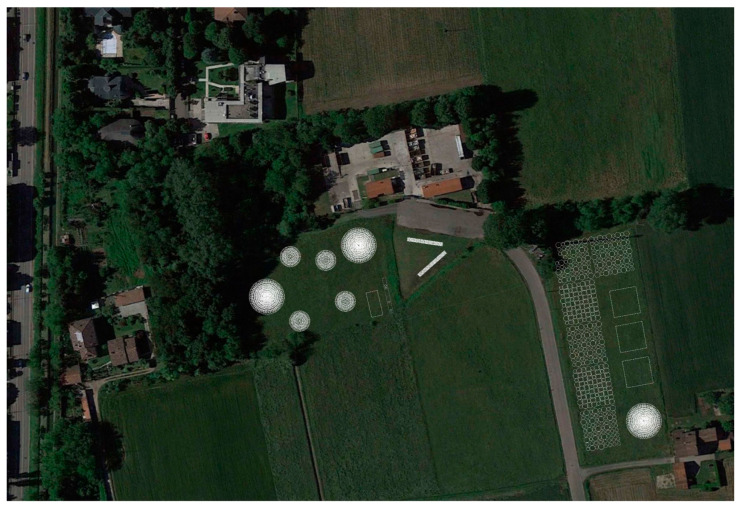
Proposed composition of the different plots and relative plantation scheme in the Municipality of Corbetta: the small white circles represent the phytoremediation plots; the big white circles, the serial maquis; the squares, the typical linear planting schemes; the lines, in the case, the ‘biodiversity strips’. (Drawings by the landscape architecture research team C. Geroldi, T. Cabai, M.U. Poli).

A multidisciplinary approach to afforestation allows for the consideration of a wide variety of aspects, including social and cultural values, the integration of the interventions into urban contexts, aesthetics and appearance, and the recreational potential of the sites involved in the project. Several areas of experimentation were addressed by integrating the expertise of landscape architecture in the process (Activity developed by C. Geroldi, M.U. Poli and T. Cabai), particularly at three sites located in the Metropolitan City of Milan (the municipalities of Abbiategrasso, Albairate and Corbetta) and Pistoia. This experience represented an opportunity to integrate contributions from different disciplines, adding value, complexity and diversity to the scientific project. These sites were approached by studying possible strategies, concepts and compositions that could accommodate the plots envisioned by scientific experimentation and respond in different ways to the contexts. The layouts foster connections between interventions and their surroundings and envision the future role of the sites after experimentation. The interventions aim to communicate their presence and the nature of scientific experimentation to passers-by or visitors through their appearance and to foster public and recreational uses when they are located near settlements. This extends the project’s impact to the social sphere and ensures durability, with an approach typical of landscape architecture that is generally overlooked in experimental projects. The site of Corbetta, for instance, has considerable recreational potential, being relatively close to a school and an urban park (Figure 4). Here, the concept consists of several circular plots in the area closer to the urban centre, envisioning the newly afforested area as a future park (Figure 5). The circular plot proved to be an interesting form to organise the space in, thus combining biodiversity and recreational uses. In contrast, the other portion of this site, more separated, was dedicated to a sequence of plots aligned with the street. The site in Abbiategrasso, close to a productive area with less recreational potential, is envisioned to be seen linearly by passers-by and workers. The selected area in Pistoia, highly visible from the highway and the city hospital, presents itself to passers-by with clear and legible geometries. The layouts were also designed to make the unique nature of the scientific experimentation legible to observers through their appearance to communicate it and foster the project’s didactic value (Geroldi and Cabai, forthcoming [39]). Indeed, the proposed strategy was to foster the communication of the scientific project to the public by making it recognisable, thus limiting natural-looking landscapes and developing an experimental project from an aesthetic perspective as well. Several visits to the site have been already organised, including a guided educational visit with a class of students of the MSc in Landscape Architecture—LLH of Politecnico di Milano. Indeed, the sites are interesting in terms of a variety of experiments connected to biodiversity, which are worth communicating to the public. In addition to the legibility of the layout and forms, a few panels were also placed at some sites (e.g., in Corbetta) to make this communication more explicit. The collaboration also involved developing drawings that could convey the changing landscape over time and permitted exchanges with the overall group during the process. For the sites located in the metropolitan area of Milan, the concepts were developed and implemented by the Regional Agency for Services to Agriculture and Forestry (ERSAF-Lombardy).

## 5. Experimental Afforestation Framework and Species Choice

### 5.1. Approach for Species Selection in a Period of Environmental Changes

Understanding forest ecology and the interactions between species and their environment is vital for making informed decisions. Global environmental changes are reshaping the ecological dynamics of forests worldwide, including those in urban areas. Rising temperatures, shifting precipitation patterns, the increased incidence of pests and diseases and the pressure of invasive alien species are altering the growth patterns, survival rates and distributions of forest communities [40,41].

Therefore, the success of urban afforestation depends on selecting species resilient to these stressors, particularly higher temperatures and extreme climate events. For instance, it is key to prioritise drought-tolerant and pest-resistant species and trees that can successfully compete with alien species [42]. Additionally, planning for initial plant diversity and designing new forests with structural diversity can foster a more resilient urban forest over time, supporting healthier forest ecosystems and reducing the risk of widespread damage from a single pest or disease outbreak while promoting a broader range of ecosystem services [42].

The selected species must not only withstand current urban conditions but also adapt to future climate scenarios, such as increased temperatures, altered precipitation patterns and more frequent extreme weather events [43]. In our project, across experimental sites, we tested several planting schemes and alternative species that are more thermophilous than those frequently used in the Italian range for temperate forests as target ecosystems. For instance, in some study areas, we have been testing the use of the thermophilous *Quercus pubescens* Willd. subsp. *pubescens* (downy oak) in place of the mesophilous *Quercus robur* L. subsp. *robur* (pedunculate oak). After only one year of observation, the species used showed a good growth and survival rate, although no differences from the other planted species have yet been observed.

### 5.2. Plantation Schemes

In this project, we tested several planting schemes (e.g., Figure 5). Besides the typical plantation scheme using about 70% tree species and 30% shrub species, we assessed the success of new and alternative plantation schemes such as the following: (1) planting more shrub species than tree species, with a reverse proportion between trees and shrubs: 70% shrub species and 30% tree species; (2) the use of successional spots of vegetation (or serial maquis), involving very dense and concentric belts (nuclei) of trees and shrub plants [44]. In particular, successional spots of vegetation (i.e., serial maquis) represent a method of afforestation that mirrors spontaneous succession. As seen in planimetry, the plantation has a concentric shape: the central part is occupied by the most evolved aspects of the vegetation dominated by tree species, while shrub species dominate the peripheral parts.

Additionally, the efficiency of planting seeds of both shrub (*Ligustrum vulgare* L. (wild privet)) and wood species (genus *Quercus* (oaks)) instead of saplings has been tested following Leverkus et al. [45]. Assessing the effectiveness of using seed tree/shrub species versus saplings can be key to determining the most effective afforestation strategy. Seeds of tree/shrub species are often used for the natural regeneration of forests due to their ability to disperse across the landscape. This method mimics natural forest regeneration, enhancing ecosystem resilience and allowing each plant individual to pre-adapt to the soil conditions (abiotic and biotic characteristics), starting from the seed stage. In contrast, planting saplings, or young trees/shrubs, generally provides faster canopy cover and growth, offering better erosion control, carbon sequestration and habitat creation. Comparative studies of these approaches might help to assess factors like survival rates, growth rates, costs and the restoration of ecological balance, informing more tailored, sustainable afforestation practices.

The long-term management (at least 5 years) and monitoring (at least 15 years) of afforestation actions (species’ survival and growth) will be assured by the participants, leveraging their interdisciplinary expertise to evaluate the ecological health of the new forests. Continuous community involvement can be fostered through participatory programmes, educational initiatives and partnerships with local stakeholders to nurture a sense of ownership.

### 5.3. Enhancing Functional Biodiversity in Newly Afforested Areas

Afforestation actions with very young plants are usually not ready to host additional biodiversity, especially animals and plants typical of forest habitats that usually require mature trees to find suitable places for their life cycle [46]. In other words, forest biodiversity and ecosystem functioning increase with forest age [47,48]. However, it is possible to accelerate the biodiversity colonisation of new forests by taking specific actions to promptly maximise local species richness and restore functional biodiversity [49], which is responsible for supporting and regulating ecosystem services. This will likely cause a direct improvement in the fitness and diversity of species, promoting complex functional relationships and preventing ecological imbalance (e.g., dominant species, parasites, exotic weeds, etc.) and the erosion of biodiversity and soil quality.

**Figure 6:**
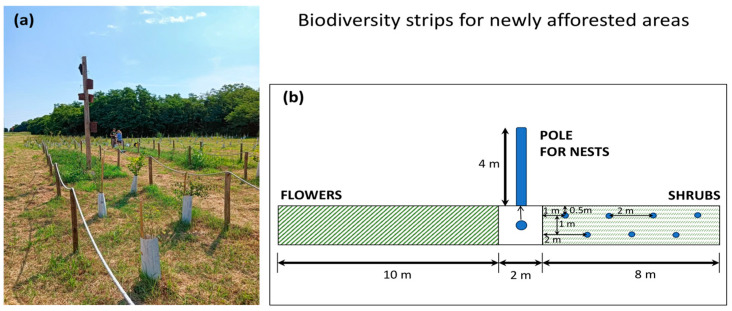
(**a**) A ‘biodiversity strip’ realised in the Municipality of Albairate (Metropolitan City of Milan) within a new afforestation area; (**b**) a schematic model of the spatial features of the strip (i.e., the relative sizes of the parts can be modified as needed) (Photography and drawing by P. Biella).

In new forests, the goal of maximising biodiversity can be achieved through targeted interventions aimed at improving habitat quality by promoting the reproduction and resource acquisition of plants and animals. Specific actions include: (1) increasing the availability of primary resources such as nectar and pollen for pollinators by planting perennial and diverse flowers, and using shrubs with berries and dry fruits for frugivorous animals, to provide nutrition to animals shortly after planting; (2) supporting primary producers with targeted inoculations of soil microorganisms and treatments with biostimulants for natural resources (e.g., water and minerals) and mitigating abiotic stress factors (e.g., high temperatures) to ensure resources are available even in challenging environmental conditions or times; (3) setting up artificial nests to host more animals, especially those providing ecosystem services (e.g., pollinators, insectivorous or seed-dispersing bats and birds) and facilitating species interactions. It must be stressed that to reactivate ecosystem functioning, enhancing actions need to target several ecosystem levels and functional groups of plants and animals: hence, it is crucial to provide a suite of measures for various types of animals and plants in recently afforested areas. Furthermore, these actions can be distributed across different places of the afforested area or concentrated in ‘biodiversity strips’: small areas dedicated to hosting actions for enhancing biodiversity. For instance, in the experimental sites in the Metropolitan City of Milan (e.g., Figure 5) and in Rome, where hectare-size areas were dedicated to novel afforestation, six ‘biodiversity strips’ measuring 20 m × 2 m were placed in each hectare. These strips have a 10 m × 2 m flowered area and an 8 m × 2 m section with four-year-old shrubs bearing berries and dry fruit, immediately ready to flower (Figure 6). Three of them host poles with nests for birds, bats and wild bees, alongside small mammal nests on the ground. By identifying and applying solutions for increasing microhabitat diversity and providing applications to readily increase functional biodiversity in newly afforested areas, ecosystem dynamics will be boosted, and young forests will host several species beneficial for ecosystem services and functioning.

## 6. Monitoring of Afforestation Interventions

### 6.1. In-Field Monitoring

Urban afforestation typically requires in-field monitoring of flora or vegetation (e.g., Celesti-Grapow and Blasi [50], Washburn [51], Rat et al. [52]), often focused on the growth and performance of planted individuals and the assessment of the evolution of plant succession, particularly with reference to native and alien species [53,54,55] and their relative traits [56]. The study of urban forests usually focuses on aspects such as forest health or forestry inventory (e.g., Mcpherson [57], Heiden et al. [58], Morgenroth and Östberg [59]), carbon storage (e.g., Myeong et al. [60]) and forest composition and structure (e.g., McPherson et al. [61], Mosyaftiani et al. [62]). A multidisciplinary monitoring approach, in which vegetation survey and forestry inventory [63] methods are used in conjunction, is sometimes applied (e.g., Veneto Agricoltura [64], Ireland’s National Forest Inventory [65]), but rarely in urban or peri-urban environments (e.g., Sitzia et al. [66], Fonge et al. [67]).

### 6.2. Past Afforestation Activities

Ecosystem succession and ecological restoration are intrinsically linked [68]. The study of successions can offer substantial contributions to the restoration of urban forests, because they provide both short-term predictions on species dynamics and a long-term perspective on biodiversity trends. The ecosystem restoration approach can, in turn, provide critical information to improve our understanding of succession in forest environments and inform practitioners about community structure (species richness, evenness, density, spatial aggregation, etc.) and community sustainability over time [68].

In particular, investigating how past afforestation projects have changed over time could help in the design of future actions. However, restoring or creating an ecosystem with a peculiar species composition can be quite difficult, especially considering the habitat structure or function in comparison with reference ecosystems [69].

Urban environments, including forest patches within them, can be involved in several types of ecological studies, for instance: (1) comparisons of different land-use types within an urban setting [70]; (2) comparisons of an urban area with a nearby ‘natural’ forested area (i.e., gradient analysis, Vizzari et al. [71]); (3) diachronic studies of urban development dynamics by monitoring a single area over time [72], etc. The in-field monitoring of past reforestations of different ages, located in various urbanisation contexts, allows for the study of urban ecosystems from multiple perspectives. These include the development dynamics of these urban ecosystems, their relationship with the urban–rural gradient and their potential for sustaining biodiversity levels and refugium areas for plants and animals [73]. These artificial ecosystems must then be compared with what could potentially be considered the ecologically ‘natural’ formations, represented by the oldest wooded formations situated in proximity to the present urban settlements in the study area and naturally colonised sites.

A study of past reforestation activities, as performed in our study, must be based on a survey of flora and vegetation in conjunction with a forest inventory, allowing for the qualitative characterisation of the formations through their physiognomic–structural traits. The investigation of these stages can provide insight into the possible evolution of the succession, as well as potential cyclical temporal fluctuations [74], and suggest what problems the new afforestation plants could encounter. Regarding the investigation of forest structure, data collection for the project considered species composition, status rating, mortality rate and tree diameter at breast height [75].

The in-field study is fundamental, considering the large variability of succession status of forest patches in man-made habitats (i.e., forest temporal dynamics). Specific restoration programmes must be based on detailed knowledge of successions in particular habitats and ecological situations [76]. It can be observed that sites that have naturally revegetated in contexts of good environmental conditions, are generally surrounded by natural vegetation, and are subject to little disturbance usually have a higher value in terms of their natural biodiversity [77]. However, in abandoned and derelict sites, the vegetation is apparently dominated by only a few species whose growth rates were less than optimal, and this does not seem to lead to an increase in biodiversity [78]. Furthermore, a comparative analysis involving naturally colonised sites could assist in identifying effective restoration strategies for abandoned sites.

### 6.3. Remote Sensing Approaches for Managing Forest Restoration

**Figure 7:**
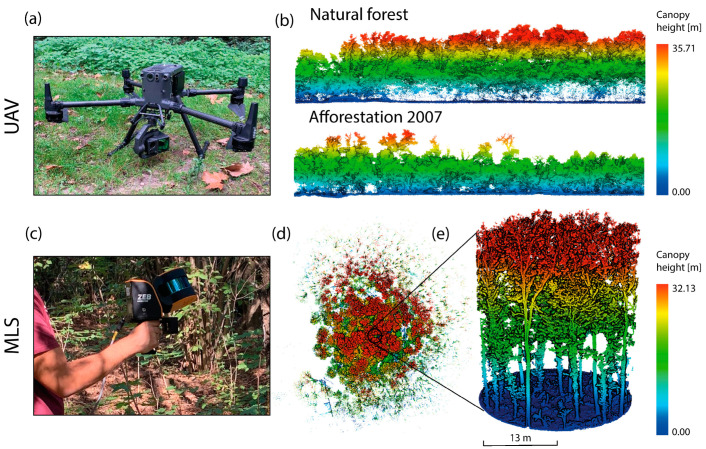
(**a**) The DJI L1 sensor mounted on the DJI Matrice 300 RTK UAV; (**b**) profile views of DJI L1 point clouds for ‘natural’ forest and reforested area in the Vanzago (MI) study area; (**c**) GeoSLAM ZEB Horizon MLS; (**d**) the top view of the point cloud acquired with the GeoSLAM ZEB Horizon MLS in the Cusago (MI) study area; (**e**) front view of 13 m radius circular plots in the same area. Point clouds are coloured according to canopy height. (Data processing and visualization by Remote Sensing of Environmental Dynamics Laboratory, Department of Earth and Environmental Sciences, University of Milano-Bicocca).

The main challenge today is quantifying the success of ecological restoration. Structural complexity is a recognised indicator of ecosystem biodiversity and, in turn, an element of restoration effectiveness [79]. In this context, monitoring using remote sensing (RS) technologies plays a key role in quantifying restoration success, due to several advantages including large-scale coverage, low operational costs and long-term monitoring. RS technologies employ active (LiDAR) or passive (optical or thermal cameras) sensors capable of capturing structural attributes for estimating structural complexity. Depending on the platforms on which they are mounted, these sensors have shown great promise in extracting tree metrics at different scales. LiDAR sensors (static, mobile or mounted on unmanned aerial vehicles (UAV) (e.g., drones; Figure 7a)) allow tree heights and crown metrics to be extracted from point clouds, capturing the vertical structure across different strata [80]. These variables often show allometric relationships with other tree metrics (e.g., diameter at breast height—DBH), acting as proxies for estimating structural complexity components [81] and Aboveground Biomass (AGB) [82]. Monitoring common sets of forest metrics using different RS techniques could provide a comprehensive and cost-effective approach for restoration assessment [79], aiding the adaptive management of new plantations. We aimed to extract the structural data of historical forests and more recent afforested areas (Figure 7b) from LiDAR data at different scales. In particular, the mobile laser scanner (MLS) system ZEB Horizon (GeoSLAM Ltd., Nottingham, UK) (Figure 7c) was used in 13 m radius circular plots (Figure 7d,e) [83], at the floristic plots following ‘star-shaped’ paths suggested by Gollob et al. [84] and Sofia et al. [85]. This system allows for the estimation of the following vegetation growth parameters: tree position, DBH, diameter, area and volume of the crowns and tree height. In our study, the raw data were first transformed into a point cloud, which was then georeferenced using the Ground Control Points (GPCs) recorded during the acquisition. The data were cleaned of noise and outliers, and clipped to the area of interest. The ground points were then classified and the point cloud normalised to obtain the heights of the scanned objects. Finally, LiDAR 360 V7 software (GreenValley International, Berkeley, CA, USA), a specific commercial LiDAR data software with a dedicated forestry toolbox, was used to segment individual trees and extract individual tree metrics (e.g., DBH and crown metrics) through its built-in functions. Other approaches may involve the use of open source software (e.g., CloudCompare (CloudCompare (GPL)) or programming languages (e.g., Python (Python Software Foundation, Wilmington, DE, USA), R (R Core Team, R Foundation for Statistical Computing, Vienna, Austria)) to extract the parameters. For example, Neudan et al. [86] explored the use of mobile laser scanning to quantify the structural complexity of forests using voxel modelling and how it varies with seasonality (presence/absence of foliage) and forest management type. The LiDAR system Zenmuse L1 (DJI Ltd., Shenzhen, China) mounted on the drone Matrice 300 RTK (DJI Ltd., Shenzhen, China) includes a Livox LiDAR module, a high-precision inertial measurement unit (IMU) and a 20 Mp RGB camera; it is installed via a 3-axis stabilised gimbal on a drone Matrice 300 RTK (DJI Ltd., Shenzhen, China) and can efficiently capture details of complex structures and provide highly accurate reconstructed models. It is also possible to visualise the captured data in real time. In this case, the parameters of interest are tree position, the height of the tree, and the diameter, area and volume of the crowns. The processing of UAV LiDAR follows the same procedure as outlined for the MLS data. UAV LiDAR data have proven to be effective in extracting tree metrics, such as detecting tree positions even in complex mixed forests [87], as well as monitoring tree height growth [88]. Good consistency between LiDAR metrics and ground-truthed data has been shown for many traditional canopy structure attributes (e.g., tree height [89], DBH [90]). However, LiDAR data offer several key advantages over traditional monitoring methods. Specifically, they enable efficient large-scale data collection, allowing for the assessment of urban forests over large areas without the need for time-intensive fieldwork. Additionally, LiDAR’s capability to monitor changes over time can aid in evaluating the effectiveness of reforestation interventions and support more informed decision-making for sustainable urban forest management.

## 7. Discussion

Under the current scenarios of global and social changes, successful urban afforestation projects require input from various disciplines such as forestry and botany, landscape architecture, urban planning and remote sensing. An inter- and multidisciplinary working team, established under the National Biodiversity Future Center in Italy, has begun to work in this direction by setting up a series of innovative actions: (1) experimental plantations at the national level across different bioclimatic regions; (2) historical landscape analysis studying past afforestation actions; (3) landscape design of the new afforestation areas; (4) using advanced monitoring tools like drones and LiDAR sensors to assess ecological connectivity and monitor forest establishment and health over time.

In particular, our research presents novel links between ecological goals, historical landscape changes and current landscape design in urban afforestation. Such a holistic approach has been rarely applied, especially in urban areas, attempting to provide a synthesis among different disciplines [91]. While scientific approaches focus on forest establishment and biodiversity enhancement over time (ecological succession), the study of forest evolution and the design activity also allow for consideration of the social and aesthetic impact on citizens and the recreational potential of the newly afforested areas. Thus, our research approach underscores the necessity of integrating afforested areas into the urban context to improve ecological connectivity [92] and public use when possible and highlights the importance of the legibility of interventions. Historical and modern landscape mapping helps guide afforestation efforts, ensuring that they not only improve biodiversity but also align with urban planning and policy. Indeed, land use and land cover change analysis facilitates the identification of key drivers behind such transformations, including urban expansion and the development of new infrastructure, often occurring at the expense of agricultural and forestry areas [93]. This process enables the recognition of trends and dynamics in land transformation, providing valuable insights for an informed decision-making process [94,95]. By considering long-term impacts, it supports the formulation of sustainable strategies and development scenarios. The findings of this study can practically contribute to the design of Green and Blue infrastructure [96] and the protection and deployment of ecological networks and corridors at a supra-local scale, with specific applications at the municipal level, also informing planning efforts for climate change mitigation and adaptation.

Another crucial component in urban afforestation that we are pursuing is the study of past afforestation actions to improve current and future forest restoration. As stressed in a recent study, this can allow afforestation success to be evaluated based not only on the planted species but also on the structure, diversity and functioning of the ecosystem [97]. Research at both the city and landscape scales reveals that urban areas can contain relatively high levels of biodiversity, and a significant percentage of species found in the surrounding natural habitat, including endangered species, has been found in urban forests [73,78,98]. The heterogeneity and diversity of forest patches (both planted or derived from spontaneous succession) found in urban areas, particularly those developed in regions where intensive farming is practised, often result in greater species richness than comparable rural areas in the surrounding region [99] with a unique ecosystem structure linked to the stresses of the urban environment [100]. For these reasons, monitoring past and current interventions is fundamental to achieving biodiversity goals and targets across urban ecosystems [101]. Conversely, it should be noted that urban forests (from spontaneous succession or derived through afforestation) are often subject to species homogenisation, making them very similar in species composition at both regional and continental scales [102].

Finally, the success of current and future afforestation projects depends on the selection of species resilient to urban stressors, including rising temperatures, shifting precipitation and invasive species [103]. Since it is predicted that climate change will increase the global risk to urban forests, the potential of species able to face climate changes might become key components in urban afforestation efforts ensuring long-term ecological sustainability [104]. For instance, the selection of heat-tolerant species, such as *Quercus pubescens* subsp. *pubescens* (downy oak) (in our experimental afforestation plots), was driven by their likely adaptability to increased temperatures and expected resilience in urban settings. Preliminary observations from the experimental plots indicate that these species exhibit good growth rates and high survival rates, but multi-year observations are needed to adequately assess the species’ performance. On the other hand, using shrubs instead of tree species may support plant survival and promote the more careful use of water resources for irrigation of forest plantations.

The afforestation of urban environments and the associated monitoring activities (including the involvement of citizens) have been reported in many cases to support urban planning, ecological services and sustainability (e.g., Chaparro and Terradas [105], Weng [106], Neyns and Canters [107]), often using mapping and monitoring methods supported by remote sensing technology (e.g., Ward et al. [108], Heiden et al. [58], Shojanoori and Shafri [109], Ciesielski and Sterenczak [110], Baines et al. [111], Jovanović and Glišić [112]). In particular, innovative remote sensing technologies like near-field LiDAR play a key role in monitoring the progress of urban forests. This allows for the precise measurement of tree heights, crown volumes and other structural features, which are important for evaluating the success of restoration efforts [79].

A critical element of the project involves ensuring the effective long-term management and monitoring of afforestation actions. The interdisciplinary team will perform long-term recording of species survival and growth to continuously assess the ecological health of the new forests. Community engagement strategies, such as participatory programmes (in part already performed) and educational activities, will be pivotal in fostering ownership and active involvement among local stakeholders [113]. Residents’ participation (local administrations and citizens) in the monitoring and maintenance of afforested areas has been promoted in different urban spaces. Earlier studies showed that people see urban woodland quality as linked to social, experiential, functional and ecological factors, with active resident participation improving both the woodland’s condition and community benefits [114].

## 8. Conclusions

Urban afforestation presents a complex challenge requiring an interdisciplinary approach. The work conducted by the working group on forest restoration of the National Biodiversity Future Center in Italy highlights the importance of combining ecological, historical, spatial quality and social goals in the design and implementation of urban and metropolitan forests. Innovative techniques, such as the use of drones and LiDAR sensors alongside fieldwork, enable monitoring the progress and success of reforestation efforts, improving ecological connectivity and sustainable urban ecosystem management. Well-planned, -designed and -monitored forests not only enhance biodiversity but also provide tangible benefits for people’s well-being.

The current stage of this project is ongoing, focusing on historical landscape assessments, landscape planning and design, the set-up of experimental plantation plots in afforestation areas (already implemented), data collection and analysis (performed for the first year), and remote sensing technologies for monitoring (in progress). Expected outcomes include the development of guidelines for sustainable urban afforestation practices, improved ecological connectivity and enhanced biodiversity within urban and peri-urban areas. Additionally, the findings aim to inform future urban planning policies and provide scalable models for ecological restoration in urban contexts.

## Figures and Tables

**Table 1:** Cities or metropolitan cities included in the study. Data from Atlante Statistico dei Comuni (ISTAT) [27] or Principali statistiche geografiche sui comuni (ISTAT) [28].

Metropolitan City	Municipalities (Involved)	Municipal Area (km^2^) [27]	Population (Number of Residents, 2022) [27]	Degree of Urbanisation [28]	Altimetric Zone [28] and Median Altitude (m a.s.l.) [27]	National Partition	Ecoregional Province [25]
Province of Campobasso	Campobasso	56.11	47,587	City (densely inhabited zone)	Inner mountain (660 m)	South	Apennine
Metropolitan City of Milan	Abbiategrasso	47.79	32,492	Towns and suburbs	Plain (120 m)	North West	Po Plain
	Albairate	14.98	4,735	Towns and suburbs	Plain (124 m)	North West	Po Plain
	Corbetta	18.69	18,819	Towns and suburbs	Plain (140 m)	North West	Po Plain
	All 133 municipalities	1,575.65	3,252,041	From City (densely inhabited zone) to suburbs	Plain (mean 122 m)	North West	Po Plain
Province of Pistoia	Pistoia	236.40	89,493	Town and suburb (medium-inhabited zone, close to Florence)	Inner mountain (360 m)	Centre	Apennine
Municipality of Rome Capital	Rome	1,287.24	2,749,031	City (densely inhabited zone)	Plain (57 m)	Centre	Tyrrhenian

## Data Availability

Data sharing is not applicable.
